# Vagus nerve stimulation: Novel concept for the treatment of glioblastoma and solid cancers by cytokine (interleukin-6) reduction, attenuating the SASP, enhancing tumor immunity

**DOI:** 10.1016/j.bbih.2024.100859

**Published:** 2024-09-17

**Authors:** Steven Brem

**Affiliations:** a University of Pennsylvania, Department of Neurosurgery, Perelman Center for Advanced Medicine, 15-141, 3400 Civic Center Blvd., Philadelphia, PA, 19104, United States; b Glioblastoma Translational Center of Excellence, Abramson Cancer Center, University of Pennsylvania, Philadelphia, PA, 19104, United States

**Keywords:** Cellular senescence, Cytokine, Glioblastoma, Immune checkpoint inhibitor, Immunotherapy, Interleukin-6, Neuromodulation, Tumor microenvironment, Vagus nerve

## Abstract

Immuno-oncology, specifically immune checkpoint inhibitors (ICIs), has revolutionized cancer care with dramatic, long-term responses and increased survival, including patients with metastatic cancer to the brain. Glioblastomas, and other primary brain tumors, are refractory to ICIs as monotherapy or in combination with standard therapy. The tumor microenvironment (TME) poses multiple biological hurdles: blood-brain barrier, immune suppression, heterogeneity, and tumor infiltration. Genomic analysis of the senescence-associated secretory phenotype (SASP) and preclinical models of glioma suggest that an exciting approach would entail reprogramming of the glioma microenvironment, attenuating the pro-inflammatory, pro-tumorigenic cytokines of the SASP, especially interleukin-6 (IL-6). A testable hypothesis now proposed is to modulate the immune system by harnessing the body's ‘inflammatory reflex’ to reduce cytokines. Vagus nerve stimulation can activate T cell immunity by the cholinergic, α7nicotinic acetylcholine receptor agonist (α7nAchR), and suppress IL-6 systemically, as well as other pro-inflammatory cytokines of the SASP, interleukin -1β (IL-1β) and tumor necrosis factor-alpha (TNF-α). The hypothesis predicts that electrical activation of the vagus nerve, with cytokine reduction, in combination with ICIs, would convert an immune resistant (“cold”) tumor to an immune responsive (“hot”) tumor, and halt glioma progression. The hypothesis also envisions cancer as an immune “dysautonomia” whereby a therapeutic intervention, vagus nerve stimulation (VNS), resets the systemic and local cytokine levels. A prospective, randomized, phase II clinical trial, to confirm the hypothesis, is a logical, exigent, next step. Cytokine reduction by VNS could also be useful for other forms of human cancer, e.g., breast, colorectal, head and neck, lung, melanoma, ovarian, pancreatic, and prostate cancer, as the emerging field of “cancer neuroscience” shows a role for neural regulation of multiple tumor types. Because IL-6, and companion pro-inflammatory cytokines, participate in the initiation, progression, spread and recurrence of cancer, minimally invasive VNS could be employed to suppress glioma or cancer progression, while also mitigating depression and/or seizures, thereby enhancing quality of life. The current hypothesis reimagines glioma pathophysiology as a dysautonomia with the therapeutic objective to reset the autonomic nervous system and form an immune responsive state to halt tumor progression and prevent recurrence. VNS, as a novel method to control cancer, can be administered with ICIs, standard therapy, or in clinical trials, combined with emerging immunotherapy: dendritic cell, mRNA, or chimeric antigen receptor (CAR) T cell vaccines.

## Highlights

• Interleukin-6 is a pro-inflammatory cytokine linked to the growth of gliomas.
• Blockade of interleukin-6 results in prolonged survival in mouse models of gliomas.
• Immune checkpoint inhibitors potentiate the inhibitory effect of cytokine reduction.
• Vagus nerve stimulation is proposed to harness cytokine reduction and treat cancer.
• Vagus nerve stimulation could block multiple cytokines to enhance immune therapy.

## Graphical abstract

**Figure:**
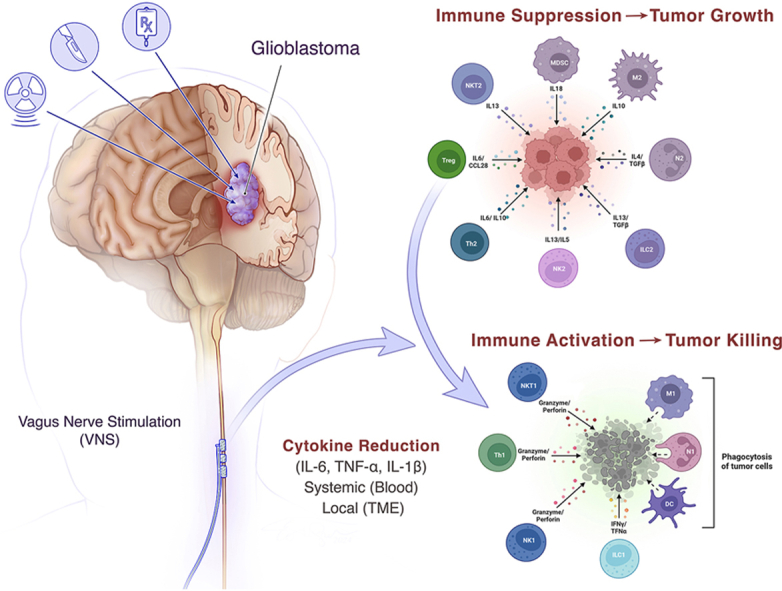

## 1 Introduction

Brain tumors are among the most fatal of cancers, with substantial morbidity [Miller et al., 2021], urgently requiring novel approaches. Gains in survival during the past four decades are primarily in younger age groups [Miller et al., 2021; Davis et al., 1998]; there have been few major advances in prevention, detection, or treatment. Specifically, 5-year survival for patients with glioblastoma only increased from 4% to 7% from 1975 to 2015 [Miller et al., 2021].

The deciphering of immune escape mechanisms has produced clinical breakthroughs in the past decade for many forms of human cancer, integrating tumor immunology into the mainstream of cancer research [Kumar et al., 2021; Finck et al., 2020; Morad et al., 2021; Propper and Balkwill, 2022]. Immune checkpoint inhibitors (ICIs) have revolutionized cancer care with dramatic, durable responses [Robert, 2020; Sharon et al., 2023], including increased survival in patients with brain metastases [Tawbi et al., 2021]. Combinations of therapies hold promise to surmount the numerous mechanisms of immune suppression in gliomas [Anderson and Reardon, 2022; Wu and Lim, 2023]. Glioblastomas and other primary brain tumors, however, remain refractory to ICIs, even when combined with standard therapy [Anderson and Reardon, 2022]. The challenge for glioma immunotherapy is to overcome the formidable genetic, epigenetic, structural, and functional barriers to immunotherapy: including the immune-resistant microenvironment, blood-brain barrier, stemness, heterogeneity and tumor infiltration along subcortical fiber tracts [Brem et al., 2023; Richardson et al., 2024]; nevertheless, glimmers of hope arise from recent reports of long-term survivors using CAR T cell [O'Rourke et al., 2017; Durgin et al., 2021] or dendritic cell [Liau et al., 2023; de Godoy et al., 2023a] vaccines.

Would it be possible to harness the power of the autonomic nervous system, to control cancer within the brain, by reversing the SASP [Coppola et al., 2014], restoring cytokine balance, attenuating inflammation (‘inflammaging’), and controlling levels of IL-6, plus other inflammatory cytokines, within the tumor microenvironment? The hypothesis presented here integrates three convergent lines of evidence to suggest a novel, feasible, theoretically sound, minimally invasive method – to convert a tumor that is immunoresistant to one that is immunoresponsive: *i*) cytokine reduction, specifically IL-6 reduction/normalization; *ii*) combination with ICI (PD-1/PD-L1 inhibition); and *iii*) vagus nerve stimulation. The objective is to validate the hypothesis in a proposed clinical trial so that the combination of these biological modifiers could keep glioblastoma in a dormant, quiescent state, “*taming glioblastoma*” [Wong and Brem, 2011; de Godoy et al., 2023b] potentially, extending this approach to other immunoresistant, refractory cancers [Finck et al., 2020].

### 1.1 Interleukin-6: the key cytokine in SASP, pathogenesis, and malignant phenotype of human glioma

Our search for a specific “signature” of glioma tumorigenesis [Coppola et al., 2014] began by exploring the genomic basis for the known link between age and the propensity to develop or succumb to a glioma. Older age is the most significant adverse prognostic factor for patients with gliomas [Coppola et al., 2014], who also develop altered immune responses with increasing age [Colopi et al., 2023]. Low-grade gliomas, for example, occur in adults with a median age of approximately forty [Mellinghoff et al., 2023]; high-grade gliomas, e.g., glioblastoma, generally occur in adults over age fifty, with a median age of sixty-two [Colopi et al., 2023]. We observed that the cellular SASP, which entails a striking increase in the secretion of pro-inflammatory cytokines [Davalos et al., 2010], including IL-6, is linked to glioma progression [Coppola et al., 2014]. The cellular “senescence score” of fourteen genes of the SASP correlated significantly with the age of the patient; IL-6 expression was linked to the patient's age, histological grade, and inversely correlated with prognosis [Coppola et al., 2014]. We suggested that the senescence-associated gene signature, including IL-6, could be used as a prognostic biomarker and a target for immunotherapy [Coppola et al., 2014]. IL-6 is highly amplified in human glioblastoma, as noted in the cancer genome atlas (TCGA) database [Coppola et al., 2014; Wang et al., 2018; Bagley et al., 2019].

**Fig. 1:**
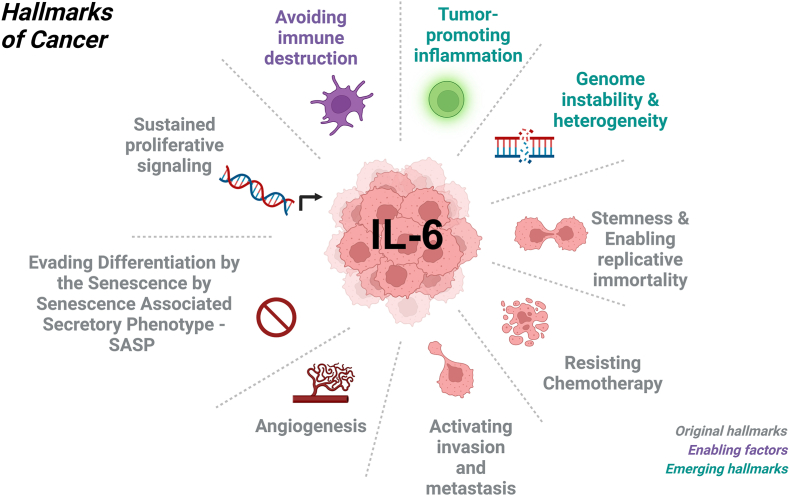
Interleukin-6 is a keystone cytokine [Hunter and Jones, 2015], linked to many of the known hallmarks of cancer [Hanahan, 2022] that comprise the malignant phenotype of glioblastoma, including *i)* angiogenesis; *ii)* chemoresistance; *iii)* heterogeneity; *iv)* immunosuppression*; v)* tumor-promoting inflammation; *vi)* invasion; *vii)* proliferation; *viii)* senescence- SASP and *ix)* stemness – making IL-6 an attractive target for cytokine reduction using VNS (Created with BioRender.com).

IL-6 itself acts as an oncogenic driver, reciprocally activating NFκB [Zanca et al., 2017; Liu et al., 2017] and is linked to many of the hallmarks of cancer (Fig. 1) [Morad et al., 2021; Hanahan, 2022] such as angiogenesis [Bonavia et al., 2011; Bikfalvi et al., 2011; Yeung et al., 2012; Shrivastava et al., 2024], stemness [Bonavia et al., 2011; Jin et al., 2012; Hossain et al., 2015; Filppu et al., 2021], invasion [Bonavia et al., 2011; Yeung et al., 2012; Saidi et al., 2009; McFarland et al., 2013], proliferation [Yeung et al., 2012; McFarland et al., 2013], heterogeneity [Zanca et al., 2017; Bonavia et al., 2011; Jin et al., 2012; Doucette et al., 2013], chemoresistance [Zanca et al., 2017; Bonavia et al., 2011; Yeung et al., 2012; McFarland et al., 2013; Filppu et al., 2021], inflammation [Morad et al., 2021; McFarland et al., 2022; Shrivastava et al., 2024], and immunosuppression [Bonavia et al., 2011; Doucette et al., 2013; Lamano et al., 2019; Yang et al., 2021], making IL-6 attractive for either targeted therapy or immunotherapy [Coppola et al., 2014] (Table 1). Importantly, not only do cytokines such as IL-6 promote local tumor growth, shaping the tumor microenvironment, but the SASP also contains chemokines that attract myeloid-derived suppressor cells (MDSCs) [Al-Kharboosh et al., 2020]. The MDSCs, through a hematopoietic, systemic circuit migrate to the tumor, and through immunosuppressive regulatory T-cells (Tregs), further augment an immune resistant environment [Salminen et al., 2018].

**Table 1:** Scientific rationale for targeting IL-6 to enhance tumor immunity.

Author, year	Findings	Implications
Weissenberger et al. (2004)	IL-6 is required for glioma development in a mouse model	Blockade of Il-6 signaling, such as VNS, could prevent malignant progression by immuno-interception, holding low-grade gliomas in a prolonged dormancy.
Brenner et al. (2007)	A single-nucleotide-polymorphism (SNP) in the IL-6 gene is linked to genetic susceptibility to develop glioblastoma	This finding supports the concept that IL-6 signaling is an oncogenic driver, and that blockade of IL-6 signaling could delay malignant progression or recurrence
Knüpfer and Preiss (2007)	Significance of IL-6 in breast cancer	Review showing that serum IL-6 levels are a strong, negative prognostic biomarker for patients with breast cancer. For example, serum IL-6 levels were found to be significantly higher in patients with breast cancer (mean 38.3 pg/ml) vs. normal women (0.7 pg/ml) (Jiang et al., 2000)
Bonavia et al. (2011)	IL-6 is linked to heterogeneity maintenance in glioblastoma	Heterogeneity is a principal cause of treatment failure in human GBM for a variety of targeted therapies and immunotherapy
Bikfalvi et al. (2011)	IL-6 is linked to angiogenesis and invasion	Blockade of IL-6 could attenuate two of the hallmarks of malignant gliomas: angiogenesis and invasion
Jin et al. (2012)	IL-6 is linked to stem cell proliferation in gliomas	“Stemness” is a key mechanism of resistance to conventional therapies such as chemotherapy, radiation therapy as well as anti-angiogenesis therapy.
Coppola et al. (2014)	IL-6 expression as part of the SASP is linked to patient's age, prognosis, and malignant progression of human gliomas	Multiple genes are part of the SASP, of which IL-6 is predominant, consistent with the role of IL-6 in inflammation, inflammaging, oncogenesis, and malignant progression, making it an ideal target for suppression via either pharmacological inhibition, immunotherapy, or as proposed, using VNS
Tsukamoto H et al. (2018)	Combined blockade of IL6 and PD-1/PD-L1 signaling abrogates mutual regulation of immunosuppression in the TME in preclinical models of glioblastoma.	Combined blockade of IL6 and PD-1/PD-L1 signals exerts a synergistic antitumor effect with increased infiltration of IFNɣ-producing CD4^+^ T cells; IL 6 is a rational immunosuppressive target to overcome the narrow therapeutic window of anti-PD-1/PD-LI therapy. This work supports the proposed combination of cytokine reduction using VNS with ICI (anti-PD-1/PD-L1).
Wang et al. (2018)	Vascular niche IL-6 induces alternative macrophage activation in glioblastoma.	In orthotopic glioma models, pharmacological blockade or gene deletion of IL-6 results in a “switch” of macrophage activation from M2-immunosuppression to M1- immunostimulation with suppression of tumor growth and prolongation of survival.
Lamano et al. (2019)	Blockade of GBM-derived IL-6 inhibits myeloid cell-derived PD-L1 and suppresses tumor growth	Systemic approach to IL-6 blockade resets immune environment and suppresses GBM growth, showing that IL-6 mediated immunotherapy does not require direct crossing of the blood-brain-barrier but strong impact in the GBM microenvironment working on myeloid cells derived from bone marrow
Ladomersky et al., 2020	Advanced age increases immunosuppression in the brain and decreases efficacy of immunotherapy in subjects with GBM	Supports the role of SASP in the pathogenesis of GBM. Senescent cells expressing the SASP result in increased immunosuppression in the brain with advancing age by proinflammatory molecules, resulting in increased survival in younger mice with GL261 orthotopic glial tumors compared to older mice, similar to the clinical situation with human glioblastoma.
Yang et al. (2021)	Dual targeting of IL-6 and CD40 is synergistic in treatment of glioblastoma	Therapeutic effects of IL-6 blockage can be enhanced by T cell activation, specifically with CD40 agonist
Hailemichael et al. (2022)	IL-6 blockade abrogates toxicity of immunotherapy and promotes tumor immunity, upregulating IL-12.	Supports IL-6 targeting, enhancing tumor immunity and efficacy while diminishing major toxicities of systemic immunotherapy. Decoupling of tumor immunity from autoimmunity (toxicity of ICI) could mean that VNS would be useful in many oncological protocols with ICI-based therapy.
Raškova et al. (2022)	Role of IL-6 in cancer invasiveness and metastasis- therapeutic opportunities	Excellent review of current pharmacological therapies targeting specific components of the IL-6 signaling pathways and evidence that IL-6 plays a key role in the pathogenesis of multiple human cancers, e.g., melanoma, ovarian, breast, NSCLC, HNSCC, and pancreatic cancer.
Baruch et al. (2023)	IL-6 blockade reduces inflammation induced by tumor-associated nerves and improves efficacy of ICI	VNS by reducing IL-6 levels could enhance the efficacy of ICIs such as anti-PD-1 therapy with broad implications for systemic cancer therapy, because anti-PD-1 therapy is already FDA-approved
Daniel et al. (2023)	IL-6 blockade (tocilizumab) inhibits GBM proliferation by suppression of neuronal-GBM circuit integration	Novel mechanism whereby IL-6 targeting could suppress GBM growth in humans by suppressing electrical activity that promotes cellular growth
Lan et al., 2023	Gliomas may evade immune surveillance by IL-6 dependent cilia-based signaling.	IL-6 recruits tumor-associated macrophages of the M2-immunosuppressive type, in part, by release from cilia in glioblastoma, and potentially other tumors, providing another IL-6 dependent process contributing to immunosuppression
Takasugi et al. (2023)	Role of IL-6 in cellular senescence and SAS in the tumor microenvironment.	Update on the key role of IL-6 in SASP, cellular reprogramming, tumorigenesis in a variety of solid cancers
Soler et al. (2023)	Update on targeting IL-6 cytokine family for cancer immunotherapy.	Excellent review of the emerging data to combine IL-6 cytokine therapy with immune checkpoint blockage and the synergistic effect on the tumor microenvironment
Yang et al. (2024)	IL-6 significantly correlates with the prognosis of LGG, while mediating the glioma immune microenvironment.	VNS potentially could be a form of immuno-interception blocking malignant progression or even transformation of LGG to HGG.
Dadgar et al. (2024)	IL-6 targeting for peritoneal carcinomatosis	The scientific rationale for targeting IL-6 in solid cancers, including advanced stages such as peritoneal carcinomatosis, and the role of IL-6 in a range of cancers, including ovarian, gastric, pancreatic, colorectal, and mesothelioma
Zhang et al. (2024)	Targeting STAT3 with nanoparticles leads to reduction of IL-6 (and IL-10) normalizes vasculature and reprograms the immune TME in GL261 glioma model	Lipid nanoparticles that cross the BBB, targeting STAT3 and IL-6 reprogram the TME, increasing a) M1-immunoresponsive TAMs, b) mature DCs, c) CD8^+^ T cells, reduction in d) Treg cells, e)M2 macrophages, and f) glioma volume resulting in an increase in survival.
Widjaja et al. (2024)	Inhibition of IL-11 signalling extends mammalian healthspan and lifespan.	IL-11 is a pro-inflammatory cytokine of the IL-6 family, recognized as having a role in cellular senescence and the SASP; blockade of IL-11, by either genetic deletion or neutralizing antibody, improved metabolism and muscle strength, reduced ageing biomarkers (e.g., frailty, mitochondria DNA and telomere length) and increased lifespan of mice from 22.5% (male) and 25% (female). Translating these results to patients using VNS, suppression of inflammation could have systemic benefits to improve lifespan and quality of life beyond focal, tumor control.

**Fig. 2:**
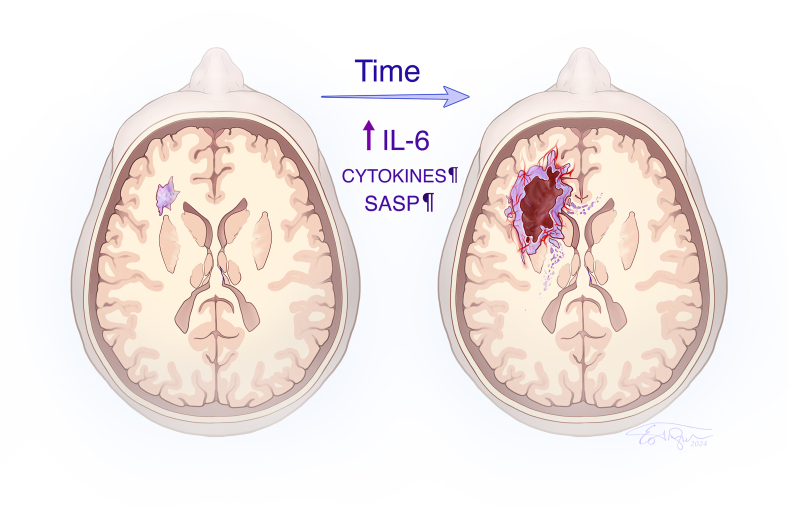
A hypothetical model of malignant progression in human glioma, with emphasis on the role of IL-6, and other inflammatory cytokines, in the development and progression of glioblastoma. The senescence-associated-secretory-phenotype (SASP) is linked to glioma pathogenesis [Coppola et al., 2014]. In panel (left), a small, residual or a low-grade glioma is shown without the hallmarks of a glioblastoma (right). A chronic, “cytokine storm” in the TME is proposed as mechanism for malignant transformation of low-grade gliomas as over 50% of low-grade gliomas convert to malignant gliomas within 5 years [Nabors et al., 2020]; The cytokine reduction hypothesis predicts that targeting of IL-6, and other companion cytokines, could thwart malignant progression, as observed in preclinical models [Wang et al., 2018; Lamano et al., 2019; Yang et al., 2021]. The hypothesis is being evaluated in the ongoing clinical trial (NRG-BN010, NCT04729959) as well as for the current, proposed clinical trial using vagus nerve stimulation. [Illustration created by Eo Trueblood].

Because the progression of low-grade (WHO II) to high-grade gliomas, or secondary glioblastoma, occurs commonly, or inevitably [Duffau, 2013; Al-Tamimi et al., 2018], carrying a dire prognosis [Jaeckle et al., 2011], an unmet need in neurooncology is to develop a preventive strategy to thwart malignant progression. More than 50% of low-grade gliomas develop tumor progression, converting to malignant gliomas within five years after surgery [Nabors et al., 2020], even after a neurosurgeon-determined gross-total resection [Shaw et al., 2008]. In a mouse model, IL-6 is required for glioma development [Weissenberger et al., 2004]. A common variant of the IL-6 gene (rs1800795 polymorphism) is linked to genetic susceptibility for development of human glioblastoma [Brenner et al., 2007]. Consistent with the putative role of IL-6 in neoplastic transformation (Fig. 2) we found IL-6 gene expression reduced in low-grade compared to high-grade (WHO IV) gliomas [Coppola et al., 2014]. Recent work supports the role of IL-6 in malignant progression of gliomas: of 146 differentially expressed genes, using TCGA-LGG data, only IL-6 emerged as an immune-related biomarker, and a therapeutic target, with a negative association between IL-6 and overall survival; low levels of IL-6 correlated with IDH1 mutation, a positive prognostic marker [Yang et al., 2024].

The essential role of IL-6 in tumor pathogenesis can be extended to other types of cancer [Mace et al., 2018; Reschke et al., 2024; Dadgar et al., 2024]. The key position of IL-6 at the crossroads of inflammation, innate and adaptive immunity, and the hallmarks of cancer (Fig. 1), provides major opportunities for novel therapy [Hunter and Jones, 2015; Jones and Jenkins, 2018; Propper and Balkwill, 2022; Dadgar et al., 2024]. Senescent stromal cells in the tumor microenvironment are sufficient to drive localized increases in immunosuppressive myeloid cells that accelerate tumorigenesis [Ruhland et al., 2016; Takasugi et al., 2023; Zhao et al., 2023]. Furthermore, stromal-derived SASP IL-6 inhibits anti-tumor T cell responses [Ruhland et al., 2016]. Overall, the accumulation of senescent stromal cells in aging patients provides a tumor-permissive, chronic inflammatory TME that shelters incipient tumor cells, allowing them to proliferate unabated by the immune system [Ruhland et al., 2016; Caetano et al., 2016]. Thus, IL-6 blockade not only has a direct effect on neoplastic cells, and the hallmarks of cancer, but also reeducates the TME towards an antitumor phenotype by altering the proportion of anti-tumor immune cells, making IL-6 an actionable target to prevent tumor progression [Caetano et al., 2016].

### 1.2 Preclinical models and first-in-human trial to target IL-6 for treatment of glioblastoma

In preclinical models, IL-6 gene deletion, or IL-6 targeted therapy, effectively blocks the growth of gliomas [Weissenberger et al., 2004; Wang et al., 2018], and synergistically in combination with anti-VEGF therapy [Saidi et al., 2009; Bikfalvi et al., 2011], or with immune checkpoint blockade [Lamano et al., 2019; Yang et al., 2021]. Yi Fan and colleagues showed that targeting IL-6, with siRNA or antibodies, prolongs survival in murine, genetically engineered, orthotopic glioma models [Wang et al., 2018]. Furthermore, they uncovered specific mechanisms including: *i*) endothelial cell (EC)-derived IL-6 induced arginase-1 expression and *ii)* immunosuppressive macrophage (M2) polarization through activation of HIF-2α, converted an immune responsive to an immune resistant microenvironment [Wang et al., 2018]. In mice bearing the ECs with IL-6 deletion, there was a 70% volumetric reduction of the tumor, and 20% of the mice showed complete eradication of tumor. IL-6 deficient mice showed the absence of pseudopalisading necrosis, a hallmark of glioblastoma, consistent with an arrest of glioblastoma (GBM) progression [Wang et al., 2018]. IL-6 deletion reprogrammed the cytokine profile of the TME, increasing the immunostimulatory, anti-tumor cytokine, IL-12, while attenuating the immunosuppressive cytokine, IL-10 [Wang et al., 2018]. Of note, IL-6 deficient mice developed normally, thus IL-6 could be pivotal in glioma pathogenesis but does not appear to be essential for normal growth and development. In a follow-up study, Yang et al. [2021] showed that the combination of IL-6 inhibition with a T cell activator, a CD40 agonist, reversed macrophage-mediated tumor suppression, sensitized the tumors to immune checkpoint blockade (ICB), and extended survival in two syngeneic GBM models, while completely eradicating GL261 tumors [Yang et al., 2021].

### 1.3 The unique tumor microenvironment of the brain

The traditional view of the brain as an ‘immunologically privileged site’ is being modified with a clear understanding of the cellular and molecular ecosystem with opposing responsive and resistant immune elements, particularly the role of the tumor associated macrophages (TAMs) in brain tumors [Sampson et al., 2020]. The myeloid-derived cells that extensively infiltrate glioblastoma, and become the TAMs, represent the most common nonmalignant cell within the TME, comprising ∼30–50% of the tumor mass [Lamano et al., 2019; de Groot et al., 2020; Sampson et al., 2020; Buonfiglio and Hambardzumyan, 2021; Nazem et al., 2022; Lan et al., 2023], now recognized as the major driver of oncogenesis, immune suppression and therapy resistance in glioblastoma [Wu and Lim, 2023]. The TAMs release IL-6, and other cytokines (e.g., IL-1β) that promote tumor progression [Buonfiglio and Hambardzumyan, 2021]. In glioblastoma, programmed death-ligand 1 (PD-L1) is the critical mediator of immunosuppression; myeloid cells in the tumor microenvironment express an elevated level of PD-L1 [Antonios et al., 2017]. Glioblastoma-derived IL-6 is necessary and sufficient for the induction of PD-L1 [Doucette et al., 2013; Lamano et al., 2019].

Overall, inflammation, the SASP, up-regulation of IL-6, and infiltration by immune cells is a hallmark of virtually all solid tumors. [Fridman et al., 2012; Domingues et al., 2016; Demaria et al., 2017; Skytthe et al., 2020; Raškova et al., 2022; Lei et al., 2023; Haldar et al., 2023; Soler et al., 2023; Takasugi et al., 2023; Burgos-Molina et al., 2024; Saha and Ettel, 2024]. Gliomas, however, have been especially challenging to treat because of their cellular heterogeneity as well as the unique microenvironment that differs from other sites in the body, specifically: 1)The TAMs within the brain are derived from two distinct populations: i) the **microglia** that arise from yolk sac progenitors, reside permanently in the brain parenchyma [Viola and Boeckxstaens, 2021], changing into an amoeboid-like shape in glioblastoma [Buonfiglioli and Hambardzumyan, 2021]; ii) myeloid-derived monocytes that circulate in the blood, invading the brain as a glioblastoma emerges and then differentiate into a **tumor-associated macrophage** [Buonfiglioli and Hambardzumyan (2021). Integrated multiomic analysis of glioblastoma shows regionally distinct myeloid cell-type populations driven by hypoxia [Sankowski et al., 2024; Wang W et al., 2024]. A notable feature in recurrent glioblastoma of patients who failed ICB is the extensive infiltration of immunosuppressive CD68^+^163^+^ (M2) macrophages [de Groot et al., 2020]. Polarization to M1 macrophages, considered to be anti-tumoral [Domingues et al., 2016; Wang et al., 2018], is promoted by upregulation of interleukin-12 (IL-12) [Domingues et al. (2016), also a result of pharmacological blockade of IL-6 (Hailemichael et al., 2022).

Tumorigenesis and molecular evolution in gliomas reflect widespread genetic and epigenetic changes that occur in a highly complex and evolving tumor microenvironment [Richardson et al., 2024]. In addition to the differences in TAMs, other unique features of the brain tumor microenvironment include the presence of: *i*) oligodendrocytes [Wang et al., 2024, Wang et al., 2024]; *ii*) myelinated white matter tracts [Brooks et al., 2021; Silver and Lathia, 2021; Salvalaggio et al., 2023]; *iii*) neural networks (connectomes) [Mandal et al., 2023; Bernstock et al., 2024; Brem and Hoch, 2024; Salvalaggio et al., 2024]; *iv*) CSF circulation and lymphatic-glymphatic drainage [Lan et al., 2023]; v) stem cell niches [Chaker et al., 2024; Sloan et al., 2024] and *vi*) the blood-brain barrier [Chacko et al., 2013; Zhang et al., 2024]. The distinct microvasculature with pericytes and a tight blood-brain barrier, as well as microglia-the CNS resident immune cells, forming resident CNS macrophages, which are viewed as the primary immune effector cells in the CNS [Domingues et al., 2016; Nazem et al., 2022; Arrieta et al., 2023; Wang W et al., 2024].

### 1.4 Translation of IL-6 targeting to a first-in-human clinical trial for glioblastoma

Taken together, the data suggest that IL-6 targeting is a highly selective, nontoxic, strategy for cancer therapy, especially glioblastoma [Wang et al., 2018]. IL-6 targeting provides a wide therapeutic window, as IL-6 is normally found in extremely low serum concentrations (<1–5 pg/ml) but rises rapidly and exponentially with systemic inflammation, as part of the innate immune response, in the μg/ml range [Hunter and Jones, 2015], a 6-log fold (1 × 10^6^) rise. The large therapeutic window suggests that IL-6 inactivation would be well-tolerated. Accordingly, we are conducting a first-in-human clinical trial [NRG-BN010, NCT04729959) for recurrent glioblastoma [Bagley et al., 2022b, Bagley et al., 2022a; Soler et al., 2023]. Patients receive the combination of anti-IL6R (tocilizumab) and ICI (anti-PD-L1, atezolizumab), as well as fractionated stereotactic radiotherapy to induce an abscopal effect designed to release tumor neoantigens, activate T cells, and stimulate their infiltration into the tumor [Ene et al., 2020]. In the clinical trial, as in the preclinical models [Mace et al., 2018; Lamano et al., 2019; Yang et al., 2021; Antonios et al., 2017; Tsukamoto et al., 2018; Kubli et al., 2021; Reschke et al., 2024], inhibition of IL6 signaling is combined with immune checkpoint blockade for their synergistic effects. Furthermore, IL6 blockade can protect patients from the autoimmune toxicity induced by checkpoint inhibitors [Stroud et al., 2019; Hailemichael et al., 2022; Zhou et al., 2023], by activating the immunostimulatory, antitumor IL-12 pathway, increasing effector Th1 and Tc1 cells in the ICI-treated tumors, while reducing the Th17 cells linked to ICI toxicity [Hailemichael et al., 2022]. Remarkably, in three large phase II/III randomized trials (Checkmate–064,–066 and −067), evaluating ICIs (nivolumab, ipilimumab), a lower IL-6 level was associated with significantly greater time of survival [Laino et al., 2020], leading to a prospective phase II trial (NCT03999749) in patients with melanoma, adding tocilizumab (IL-6R blocking antibody) in combination with ICIs, nivolumab and ipilimumab [Laino et al., 2020].

Further evidence that IL-6 blockade is synergistic with ICI is to be found in a melanoma model, where IL-6 blockade widens the therapeutic window of ICI; PD-1 or PD-L1 blockade, by itself, increases systemic production of IL6 as well as stimulating PD-1^+^ macrophages to produce IL-6 in the TME [Tsukamoto et al., 2018]. Combining IL-6 blockade with PD-1/PD-L1 blockade results in a vigorous tumoricidal T-cell response, producing IFNγ-, revealing a synergistic crosstalk between the PD-1/PD-L1 and IL-6 signaling pathways [Tsukamoto et al., 2018; Soler et al., 2023]. Lamano et al. [2019] showed that human glioblastoma-derived IL-6 stimulated myeloid cells to express PD-L1 in a STAT3-dependent mechanism; inhibition of IL-6 signaling in orthotopic murine glioma models was associated with reduced myeloid PD-L1 expression, diminished tumor growth, and increased survival.

The metabolic profile, and dynamic plasticity, regulate the expression of immune checkpoint molecules [Saha and Ettel, 2024]; TAMs have become important targets for cancer therapy [Domingues et al., 2016; Wang et al., 2018; de Groot et al., 2020; Lee et al., 2021; Saha and Ettel, 2024; Wang W et al., 2024]. Further supporting the synergies of IL-6 blockade and ICB is the finding that inflammation induced by tumor-associated nerves promotes resistance to anti-PD1 therapy in cancer patients and is targetable by blockade of interleukin-6 [Baruch et al., 2023].

### 1.5 Cancer neuroscience: the cytokine and neural influence over cancers outside of the CNS

Importantly, the neural influence over cancers outside of the CNS is becoming increasingly recognized [Mauffrey et al., 2019; Amit et al., 2024]. In addition to the cholinergic influence of the vagus nerve, the sympathetic nervous system can influence cancer growth [March et al., 2020; Guillot et al., 2022]. Adrenergic, sympathetic innervation, for example, can activate an angiogenic switch to transition the growth of prostate cancer from innocuous dormancy to a high-grade malignancy [Zahalka et al., 2017; March et al., 2020].The modulating role of peripheral nerves to influence cancer growth and metastasis, first demonstrated in prostate cancer, extends to other cancers, including gastric, pancreatic, and non-melanoma skin cancer [March et al., 2020; Guillot et al., 2022]. In a murine model of pancreatic adenocarcinoma, sympathectomy promotes cancer progression, metastases, and decreased survival, linked to the influx of pro-tumoral CD163+ macrophages [Guillot et al., 2022].

Scientific evidence unequivocally links cancer progression in mice to the presence of inflammatory cytokines such as IL-6, IL-1β, TNFα, and others [IL-10, TGFβ) [Liu et al., 2021; Lei et al., 2023]. Chronic inflammation, a hallmark of cancer within and outside of the CNS, is orchestrated by numerous cell types (myeloid-derived TAMs, MDSCs, APCs, DCs, T cells, progenitor cells) and modified by local variations of the tumor microenvironment. For example, chronic inflammation drives the growth of colorectal cancer through the dysregulation of molecular pathways in the immune system; infiltration of macrophages results in the release of IL-6 (and IL-17, TNFα) fostering tumor proliferation and invasion [Burgos-Molina et al., 2024]. IL-6 molecular signaling in inflammation also involves sub-components such as receptor (IL-6R), gp130, and coordinate STAT3 and NF-κB pathways [Raškova et al., 2022; Liu et al., 2017]. IL-6 as a therapeutic target to suppress cancer cell invasiveness and metastases is supported by data from numerous cancers including pancreatic, head and neck, breast, ovarian, non-small-cell lung cancer, and melanoma [Raškova et al., 2022].

### 1.6 The vagus nerve, the “inflammatory reflex” and neural modulation of cytokines including IL-6

Our current concepts linking aging, cellular senescence, and inflammation (“inflamm-aging”) with tumor progression [Davalos et al., 2010; Coppola et al., 2014]; or melding oncology with immunology [Robert, 2020; Wu and Lim, 2023] generally overlook the vast potential of the immune system functioning with the nervous system as two intertwined “*supersystems”* [Elenkov et al., 2000]. Pioneering work by Elenkov et al. [2000] and Tracey [2002] shed light on the capability of both the sympathetic and the parasympathetic components of the autonomic nervous system to modulate the cellular elements (e.g., macrophages, lymphocytes) of the human innate and adaptive immune response. In particular, the seminal finding of the “*inflammatory reflex*” [Tracey, 2002; McAllen et al., 2022], [Table 2] demonstrated that inflammation as a physiological process can be regulated by the nervous system, mediated by the vagus nerve and the parasympathetic (cholinergic) fibers, in “real time”, analogous to the iterative, dynamic neural control over cardiac rhythm, respiration, and other vital functions [Borovikova et al., 2000; Tracey, 2002; Wang et al., 2003; Tracey, 2007; Rosas-Ballina et al., 2011; Andersson and Tracey, 2012; Koopman et al., 2016; Genovese et al., 2020; Datta-Chaudhuri et al., 2021; Natarajan et al., 2024].

**Table 2:** Scientific Rationale for using Vagus Nerve Stimulation to Target Inflammation and Cytokines, including IL-6, for Control of Cancer Growth including Glioblastoma.

Author, year	Findings	Implications
Borovikova et al. (2000)	First report that VNS attenuates systemic inflammation – in response to endotoxin.	Seminal observation linking the vagus nerve, and its stimulation, to control of systemic inflammation
Tracey (2002)	Describes the “inflammatory reflex”	The link between the vagus nerve and systemic inflammation is foundational for potential treatment of human diseases linked to inflammation, aging (inflammaging), and the SASP, ranging from arthritis to cancer
Wang et al. (2003)	Discovery of the nicotinic acetylcholine receptor (α7nAChR) and the link of a neurotransmitter to inflammation.	Electrical stimulation of the vagus nerve reduced cytokines of the SASP [IL-6, IL-1β, and TNFα]. The link between a neurotransmitter and T cells provides a strong biological rationale for using VNS in cancer immunotherapy
Koopman et al. (2016)	VNS inhibits cytokine production and disease severity in rheumatoid arthritis.	The scientific basis for the current ReSet-RA trial (NCT04539964), showing that pro-inflammatory cytokines (IL-6, IL-1β, and TNFα) are significantly suppressed by VNS.
Sullivan et al. (2018)	Young women with coronary artery disease exhibit higher concentrations of IL-6 and in response to mental stress	Plasma levels of IL-6 were between 1 and 2 pg/ml and rose with stress to as high as 4 pg/ml, especially in younger women.
Genovese et al. (2020)	Safety and efficacy of VNS in patients with refractory rheumatoid arthritis (RA) (NCT03437473).	Safety established for VNS to treat medically refractory RA with preliminary evidence of efficacy, setting the stage for a larger, sham-controlled RCT (RESET-RA; NCT04539964)
Bremner et al. (2020)	Transcutaneous VNS (tcVNS) blocks stress-induced activation of IL-6 in PTSD in a double-blind, sham-controlled RCT	Baseline IL-6 levels (blood) were <1 pg/ml (mean) with an elevation with stress-induced tasks to as high as 4 pg/ml; these levels were attenuated with tcVNS. IL-6, IL-1β, and TNFα selected as biomarkers of inflammation.
Reijmen et al. (2021)	Transcutaneous VNS stimulates tumor infiltrating lymphocytes, activates dendritic cells, arrests the proliferation of myeloid-derived stem cells (MDSCs) in preclinical models of lung cancer	VNS could be used to optimize tumor responses for patients with cancer outside of the CNS. Tissue analysis of immune correlates was more informative than immune monitoring of peripheral blood. Of note, tc-VNS may be weaker than minimally invasive VNS, and the stimulation parameters (optimal frequency, duration and strength) were not evaluated nor was VNS combined with immune checkpoint blockade.
Zhang et al. (2021)	Activating the vagus nerve regulates inflammatory cytokines, enhances tumor immunity in mice with breast tumors.	VNS could be effective potentially for human breast cancer. Clinical trials seem warranted.
Jarmuzek et al. (2023)	Cytokine profiles in glioblastoma development vs. healthy individuals.	The blood concentrations of IL-1β, IL-6, and TNF-α confirm that inflammation is a critical component of GBM development. Baseline levels of IL-6 were elevated in patients with GBM (mean, 22.5 pg/ml) vs. healthy controls (16.25 pg/mL). A GBM-specific molecular signature was proposed using levels of IL-6 and IL-1β.
Huang et al. (2023)	Crosstalk of nervous and immune systems in pancreatic cancer.	Excellent review of cancer neuroscience, including the role that acetylcholine released by the vagus nerve exerts its effect through the α7nAChR found on macrophages, inhibiting the production and release of pro-inflammatory cytokines such as TNF, IL-1β and IL-6.
Abdullahi et al. (2023)	Non-invasive VNS could be used to bolster cancer therapy and immunotherapy	A hypothesis-review paper focusing on the role of VNS to potentiate immunotherapy and cancer therapy, focusing on the cholinergic, “inflammatory reflex”, and the hypothalamic-pituitary axis, as well as oxidative stress via SIRT1. In contrast to the current hypothesis, these authors did not focus on cytokines, such as IL-6, glioblastoma, or the key role of ICI, as well as neurosurgical, minimally invasive VNS. These authors, however, note that VNS could provide additional clinical benefits: reduced lymphopenia, improvement of fatigue, and enhanced quality of life, making it especially attractive for the elderly population.
Kumaria and Ashkan (2023)	VNS could block glioma growth through glutamine neurotransmission	The recently discovered glial-glioma synapses mediated by glutamine have fostered potential therapies exploiting “cancer neuroscience” and the opportunity to repurpose electrical neuromodulation to block glutamine-driven pathways of glial cell proliferation, including DBS (deep brain stimulation), VNS, electric field therapy, as well as pharmacological inhibition of glutamate pathways.
Bernstock et al. (2024)	Neurosurgical intervention in cancer neuroscience	Broad overview of the emerging field of cancer neuroscience, connectomal-based glioma surgery, as well as the potential role of neuromodulation, including the use of VNS to reduce inflammation and oxidative stress.
Peterson et al. (2024)	Initial results of the RCT RESET-RA study (NCT04539964)	Safety established, efficacy data pending using VNS for RA. Stimulation parameters are 10 Hz pulse frequency, 0.25 ms pulse width, and 60-s pulse train duration, once daily, based on preclinical, animal models of inflammation (Levine et al., 2020).
Natarajan et al. (2024)	VNS ameliorates inflammation in a rat model of multiple sclerosis	Proof of principle that VNS could be used to control inflammation within the CNS, and potentially be a novel treatment for demyelinative disease.

At the intersection of immunology and neuroscience, the reflex neural circuit mechanism that regulates innate and adaptive immunity provides an extraordinary opportunity to impact human disease where inflammation is a cornerstone [Koopman et al., 2016]. The initial discovery in animal models (mouse, rat, and dog) found that electrical stimulation of the vagus nerve significantly reduced cytokines (IL-1β, TNF-α, and IL-6) linked to inflammation and the SASP due to the production of acetylcholine, a cognate ligand for α7nAChR [Wang et al., 2003], expressed on cytokine-producing monocytes, macrophages, and stromal cells [Andersson and Tracey, 2012; Koopman et al., 2016]. The ligand binding of acetylcholine inhibits the nuclear translocation of NFκB signaling axis, and inhibits inflammasome activation in macrophages otherwise activated by proinflammatory stimuli [Koopman et al., 2016]. In patients with epilepsy, a single stimulus of the vagus nerve leads to a significant drop in the three major cytokines of the inflammatory response and the SASP (IL-6, TNF-α, and IL-1β) [Koopman et al., 2016]. The safety and efficacy in reducing serum cytokines led to a preliminary study of 18 patients with rheumatoid arthritis (RA) in which VNS resulted in a significant drop in IL-6 levels and clinical improvement in both study groups: *i)* a treatment-naïve group with newly diagnosed RA; *ii*) a treatment-refractory group that failed standard treatments for RA, including methotrexate and two or more biological agents with distinct mechanisms of action, e.g., anti-TNF, anti-IL6R, or anti-CD20. There were no serious adverse events, no infections, and only the anticipated, mild to moderate side effects of VNS surgery [Koopman et al., 2016].

## 2 Hypothesis

**Fig. 3:**
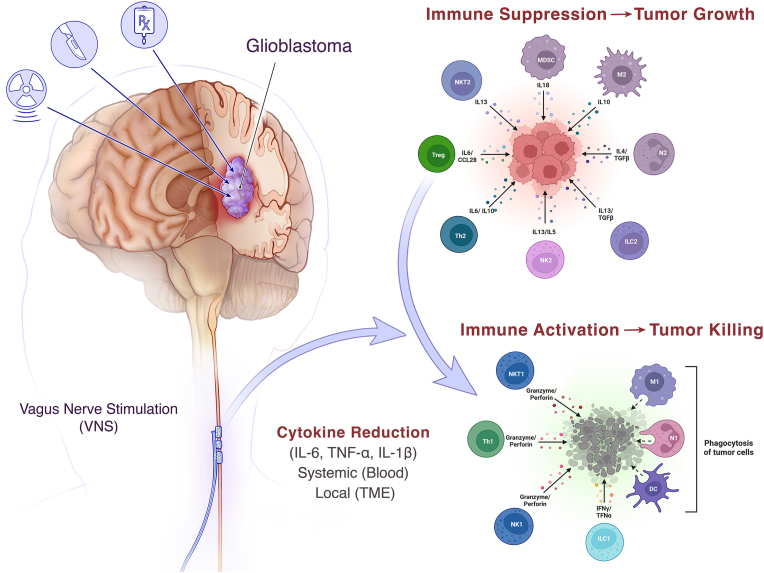
The Vagus Nerve –Tumor Immunity Hypothesis. A hypothesis is proposed that the immune system, modulated by the ‘inflammatory reflex’ [Tracey, 2002; Levine et al., 2020], activated therapeutically by VNS acting as a “master switch”, will suppress systemic inflammation, mediated by inflammatory cytokines (mainly IL-6, but also other pro-inflammatory cytokines, e.g., IL-1β, TNF-α) to provide a systemic therapeutic approach to cancer, by cytokine normalization. Illustrated is a tumor (purple) within the CNS (glioblastoma), whereby cytokine reduction converts an immunosuppressive, “cold” TME (top, right) to an immune responsive, tumoricidal environment (bottom right), marked by polarized M1 macrophages, stimulated T cells, activated dendritic cells, and immunostimulatory cytokines (e.g., IFNɣ). The integration of two major biological systems extends the concept of “cancer neuroscience” using a minimally invasive tool, VNS to reset the immune “rheostat”, reducing levels of IL-6, thereby removing a major barrier to immune eradication of cancer. Cytokine reduction with VNS can be given in combination with ICI to be maximally effective, and potentially used synergistically with other immunomodulatory approaches, e.g., vaccines (dendritic cell, CAR T cell, mRNA), viral oncolytic therapy, or anti-angiogenic therapy. The nervous system and the immune system normally (physiologically) work in harmony (homeostasis), but pathologically (allostasis) in opposition through inflammation, promoting glioma growth and tissue (neurological) injury (red) in the surrounding white matter, neuropil, and TME. [Illustration created by Eo Trueblood]. (For interpretation of the references to color in this figure legend, the reader is referred to the Web version of this article.)

Based on the preceding lines of evidence, a hypothesis is proposed that the immune system can be activated locally and systemically by suppression of IL-6 (and related cytokines, IL-1β, TNF-α) using VNS. Activating the “inflammatory reflex” by VNS for cancer control, as with immunomodulation for rheumatoid arthritis, will predictably transform the immunosuppressive TME. The hypothesis predicts that blockade of IL-6 and its companion cytokines, TNF-α, and IL-1β, will convert an immunologically “cold” (unresponsive) tumor to one that is “hot” (immune responsive). The hypothesis views glioblastoma as a pathophysiological disorder of two biological systems (the nervous system and the immune system). These systems normally (physiologically) work in harmony (homeostasis), but pathologically (allostasis) are in opposition through inflammation, promoting glioma growth and neurological injury. The imbalance of cytokines, cell growth, immune dysfunction, and CNS control proceeds at multiple levels (Fig. 3).

## 3 Evaluation of the hypothesis: future directions

The best method to evaluate the hypothesis is a proposed, scientifically rigorous, phase II trial [Table 3] that would use VNS, exploiting the “inflammatory reflex,” to reduce IL-6 levels systemically, and in combination with ICI, create an immune responsive TME in patients with glioblastoma. Overall survival [Liau et al., 2023; Hotchkiss et al., 2023] and tumor response [Bagley et al., 2022a; Bagley, 2023; de Godoy et al., 2023a] are important endpoints. The proposed trial using VNS for patients with glioblastoma; the mechanism of ‘cytokine reduction’ for glioblastoma, is innovative. Vagal nerve stimulation received FDA-approval for the treatment of epilepsy in 1997 [Fridley et al., 2012; Johnson and Wilson, 2018; Jung et al., 2023] and for treatment of depression in 2005 [Shuchman, 2007; Jung et al., 2024]. In addition to the current standard of neurosurgical debulking or “cytoreduction” [Domino et al., 2020; Patrick et al., 2022; Hajtovic et al., 2023; Chang et al., 2024], the hypothesis opens the way to evaluate “*cytokine reduction*” as a novel treatment modality for glioblastoma. Cytokine reduction would complement current FDA-approved, NCCN-endorsed [Nabors et al., 2020] modalities of maximal safe resection (cytoreduction); radiation therapy; and temozolomide chemotherapy [Stupp et al., 2005] (Fig. 3). Addition of tumor-treating fields [Stupp et al., 2017] could be considered as part of a novel therapeutic strategy targeting glutamatergic neurotransmission [Kumaria and Ashkan, 2023] but not simultaneously with VNS, as either modality likely stimulates similar or identical neural circuits using distinct populations of vagal neurons [Jin et al., 2024].

**Table 3:** Future directions to develop vagus nerve stimulation and cytokine (IL-6) reduction for cancer therapy.

#	Questions/Aims	Proposed Solution/Approach	Comments	References
1	Can we suppress growth and increase overall survival for patients with **newly diagnosed GBM**?	Combine standard of care (Fig. 3) with cytokine reduction (IL-6, IL-1β, TNF-α) and ICI (PD-1/PD-L1 inhibitor) using VNS in a phase I/II trial, “*RESET-GBM*”	Based, in part, on the RESET-RA trial (NCT04539964) using VNS as a biological response modifier to reduce IL-6; also based on NRG-BN010 (NCT04729959), using safety run, targeting IL-6 (tocilizumab) with PD-L1 inhibition (atezolizumab) for recurrent GBM	Coppola et al. (2014) Koopman et al. (2016) Wang et al. (2018) Genovese et al. (2020) Bagley et al. (2022a) Brem (2023) Peterson et al. (2024)
2	Is there a role for cytokine reduction (IL-6, etc.) to “intercept” malignant transformation of **low-grade glioma?**	Randomized controlled trial of patients with low-grade glioma and VNS compared with sham control, combined with best standard of care (Fig. 2) plus vorasidenib.	Compelling evidence that levels of IL-6 are correlated with overall survival for patients with low-grade glioma, and data from TCGA and studies on the SASP linking IL-6 to malignant progression. High rate of progression despite surgical resection.	Weissenberger et al. (2004) Shaw et al. (2008) Coppola et al. (2014) Caetano et al. (2016) Mellinghoff et al. (2023) Yang et al. (2024)
3	Is there a role for cytokine reduction- VNS in the treatment of **recurrent glioblastoma?**	A randomized phase II trial for recurrent GBM with and without tocilizumab to follow NRG-BN010.	There remains a large unmet need for treatment of rGBM. Prevention of recurrence of GBM is linked to long-term survival.	Bagley et al. (2022b) Hertler et al. (2023)
4	Is there a role for cytokine reduction using VNS in the treatment of **systemic cancer** resistant to ICI?	Based on the data and hypothesis presented here, VNS could be broadly applied to a wide range of human cancers refractory to standard therapy, targeted therapy, and/or immunotherapy. A phase I/II trial as proposed for glioblastoma could be applied to refractory NSCLC, breast, colorectal, pancreatic cancers and melanoma.	Inflammation is recognized as a hallmark of the malignant phenotype. In combination with ICIs, vagal nerve stimulation could be a new approach to numerous solid cancers that are refractory to standard therapy. Durable responses are found in only 20% of patients receiving ICIs, for the remainder the combination of ICI with IL-6 reduction through VNS could be a possible option.	Hailemichael et al. (2022) Hanahan (2022) Berland et al. (2024)
5	Is there an actual **“set point”**, a level of IL-6, and other cytokines that tilts the “immuno-stat” from immunoresponsive to immunoresistant?	In the proposed trials, it will be important to have a baseline, serum IL-6 level, and stratify outcomes based on that level as there is great variation. The object will be to “normalize” the serum Il-6 level to a value between 0 (non-detectable) to 7 pg/ml, and stratify patients in 4 groups: a) 0–2 pg/ml; b) 2–10 pg/ml; c) 11–100 pg/ml and d) > 100 pg/mL.	High serum IL-6 levels are linked to poor outcomes for numerous types of cancer. For glioblastoma, a value of 19.8 pg/ml was linked to a three-fold higher chance of GBM development. In patients with rheumatoid arthritis treated with VNS, the responders had serum level of IL-6 at 5.0 ± 1.4 pg/ml compared with an elevated level of 15.4 ± 2.4 pg/mL in clinical non-responders.	Koopman et al. (2016) Sullivan et al. (2018) Bremner et al. (2020) Laino et al. (2020) Jarmuzek et al. (2023)
6	Apart from serum monitoring of pro-inflammatory cytokines, how can the effects of **cytokine reprogramming of the TME be monitored and measured?**	“Window of opportunity” (WoO) trials with sampling and immunophenotyping of the tumor microenvironment before, during, and after VNS will be important, with longitudinal serum and tissue sampling.	Longitudinal, direct tissue sampling is more feasible for tumors outside of the CNS due to safety concerns, but WoO trials are being considered for glioblastoma. Decreased inflammatory signaling potentiates PD-1 immunotherapy for patients with non-small cell lung cancer (Mathew et al., 2024)	Antonios et al. (2017) O'Rourke et al. (2017) Tsukamoto et al. (2018) Huang et al. (2019)de Groot et al. (2020) Lee et al. (2021) Singh et al. (2022) Baruch et al. (2023) Riviere-Cazaux et al. (2023) Singh et al. (2023) Bagley et al. (2024b) Chen et al. (2024) Everson et al. (2024) Mathew et al. (2024) Wang L et al. (2024)
7	Are there blood, neuroimaging, or AI/ML-based, **biomarkers of the immune TME?**	“Liquid biopsies”, advanced imaging, including MRI-based “connectomics”, PET scanning, as well as AI/ML-based databases are being developed and could be incorporated in a VNS-cytokine reduction study to treat GBM or systemic cancer.	White matter tract density could correlate with overall survival in GBM and be a MRI-measure of the immune TME (Salvalaggio et al., 2023). The integration of PET/MRI with liquid biopsy shows promise (Khalili et al., 2023).	Hachem et al. (2018) Salvalaggio et al. (2022); de Godoy et al. (2023b) Khalili et al. (2023) Riahi Samani et al., 2023 Salvalaggio et al. (2023) Stoecklein et al. (2023) Cotter et al. (2024) Guo et al. (2024) Reschke et al. (2024) Uslu et al. (2024) Zirem et al. (2024)
8	Would VNS-Cytokine Reduction be used in a **Combination Immunotherapy Protocol**?	As chronic inflammation and pro-inflammatory cytokines (IL-6, TNF-α, IL-1β) are drivers of the malignant phenotype, cytokine reduction with VNS may be valuable but by itself insufficient to control cancer growth, without also using an immune checkpoint inhibitor and a method to activate T cells to infiltrate into the tumor. Suppression of IL-6 can be used in combination with a checkpoint inhibitor and a CD40 agonist to activate T cells.	VNS with cytokine reduction could be used to enhance efficacy of next-generation vaccine trials for GBM and other solid cancers, e.g., dendritic cell, CAR T cell, and mRNA-based vaccines.	Mace et al. (2018) Finck et al. (2020) Kubli et al. (2021) Kumar et al. (2021) Lee et al. (2021) Yang et al. (2021) Bagley et al. (2022a) Hailemichael et al. (2022) Wu and Lim (2023) Liau et al. (2023) Propper and Balkwill (2022) Bagley et al. (2024a) Berland et al. (2024) Mendez-Gomez et al. (2024) Uslu et al. (2024)
9	What would be the **electrical parameters, dosing, frequency** for the RESET-GBM trial?	First, to establish safety, a standard 3 + 3 design [Bagley et al., 2022b, Bagley et al., 2022a; Ahluwalia et al., 2024]to “dose” escalate to a maximal tolerated dose, simultaneously monitoring reduction in serum, pro-inflammatory cytokines (IL-6, TNF-α, IL-1β), then to maintain for at least one year, as VNS is designed to be a “biological response modifier” and treat chronic inflammation. The parameters based on the current RESET-RA (NCT04539964) should be a safe starting point. Combining with an ICI, as in NCT04729959 [NRG BN010], is important to reprogram the immune TME. Designing also a WoO, neoadjuvant arm will be invaluable to perform immune profiling on the TME.	The stimulation parameters (10 Hz pulse frequency, 0.25 ms pulse width, 60-s pulse train duration, once/day) are based on extensive preclinical studies (Levine et al., 2020) and designed to decrease systemic inflammation by activating the cholinergic, anti-inflammatory pathway (Tracey, 2007). Once established that inflammation can be consistently, effectively, and safely suppressed in this population (cancer patients, glioblastoma), it is eventually conceivable that the frequency and intensity of VNS could be adapted to the individual patient to effect personalized, precision medicine. Patient stratification, identification of relevant biomarkers, and appropriate stimulation parameters is the pivotal next step to move VNS forward to widespread implantation in the clinic (Schiweck, 2024)	Tracey (2007) Koopman et al. (2016) Levine et al. (2020)de Groot et al., 2020 Bagley et al. (2022a) Bagley et al. (2022b) Kelly et al. (2022) Jarmuzek et al. (2023) Bagley (2023) Ahluwalia et al. (2024) Peterson et al. (2024)
10	What future advances are being developed in **cancer neuroscience** and **bioengineering**?	Further studies will elucidate the distinct vagal neurons that modulate the immune response throughout the body with enormous implications for therapeutic neuromodulation of cancer and other diseases amplified by inflammation (Jin et al., 2024). Using optogenetics, the dorsal motor nucleus is shown to be a key hub regulating inflammation (Falvey et al., 2024) Newer designs of electrodes include optimized high-frequency, microburst VNS (Drees et al., 2024) that can be guided by fMRI (Szaflarski et al., 2024) and neuromorphic electro-stimulation (Bao et al., 2024)	Neuromorphic electrical stimulation minimizes nerve damage and may be more effective (73.5% decrease) to reduce levels of the inflammatory cytokine, IL-6 (Bao et al., 2024)	Jin et al. (2024) Falvey et al. (2024) Bao et al. (2024) Szaflarski et al. (2024) Drees et al., 2024

A benefit of VNS is that a single modality reduces not only a single target, IL-6, but also a group of inflammatory cytokines such as TNF-α and IL-1β, molecules working in concert. With IL-6 as the dominant cytokine, the suppression of TNF-α and IL-1 ß, could prove to be more effective than a monoclonal antibody that precisely targets a single cytokine. VNS could overcome some of the major reasons why cytokine monotherapies fail: *i*) short-half-life in the circulation; *ii*) pathway redundancy of the target (i.e., multiple other proinflammatory cytokines would subsume the role of IL-6); *iii*) context-dependent and *iv*) pleiotropic effects, and poor biodistribution [Propper and Balkwill, 2022]. Targeting only a single cytokine could contribute to immune resistance or escape, as the other cytokines then restore an immunosuppressive TME. Vagus nerve stimulation, by targeting a key phenotype (inflammation) and multiple companion cytokines in addition to IL-6 [IL-1β, TNFα, etc.] could be broadly applied to multiple types of cancer to combat immune evasion and cytokine redundancy [Soler et al., 2023] [Table 1].

Using serum IL-6 levels as an endpoint could provide a targeted biomarker, a therapeutic and diagnostic endpoint (theranostic), in ‘real-time’ to evaluate the efficacy of VNS to suppress inflammation, and potentially adjust the electrical parameters or frequency of stimulation. A dynamic ‘real-time’ biomarker would be welcome to evaluate response to therapy of gliomas due to the difficulty of interpreting MRI findings of progression from treatment-related necrosis, “pseudoprogression” [de Godoy et al., 2023b]. In addition, markers of inflammation, C-reactive protein (CRP), neutrophil – lymphocyte ratio (LNR), the CRP–albumin-lymphocyte (CALLLY) index could be used as an index of inflammation, as well as a serum cytokine panel, (e.g., IL-6, IL-1β, TNF-α) to detect the effect of VNS on coordinate cytokines and suppression of systemic inflammation [van Sleen et al., 2023; Yang et al., 2023; Cotter et al., 2024].

VNS is being evaluated in clinical trials for numerous disorders, ranging from specific “neurological diseases”, i.e., spinal cord injury [Fallahi et al., 2023], and stroke [Korupolu et al., 2024] to systemic, chronic “inflammatory diseases” [Bonaz et al., 2016; Johnson and Wilson, 2018], including depression [Steffen et al., 2024] metabolic syndrome [Das, 2011], rheumatoid arthritis [Van Maanen et al., 2009; Koopman et al., 2016], and Crohn's Disease [Levine et al., 2020]. Advantages to the use of VNS include established safety, and minimal side effects based on a vast clinical experience in refractory epilepsy and depression [Das, 2011]. VNS could provide additional benefits to patients with glioblastoma for whom seizures and depression are common and serious setbacks [Walbert and Stec, 2023]. Increasing evidence corroborates the fundamental role of neuroinflammation in the development of epilepsy with elevated levels of IL-6 and companion cytokines, IL-1β and TNF-α [Soltani Khaboushan et al., 2022]. Up to 80% of patients with a glioma experience one or more seizures during their disease course; for patients with glioblastoma, the rate is lower, 30–50%, less than patients with lower-grade gliomas [Sokolov et al., 2023] but still a substantial, clinical problem.

Nearly 90% of patients with a malignant brain tumor develop depression, significantly more common than patients with other types of cancer, <30% [Finze et al., 2023]. Anti-inflammatory agents reduce depressive symptoms, especially in patients with autoimmune and inflammatory disorders [Kappelmann et al., 2018; McFarland et al., 2022; Steffen et al., 2024]. Systemic biological, anti-cytokine agents show a significant improvement of symptoms of depression in patients with chronic inflammatory disorders, specifically targeting IL-6 (tocilizumab) or TNF-α (adalimumab, etanercept, infliximab) [Kappelmann et al., 2018]. Thus, VNS could be valuable to improve the quality of life as well as extending survival by tumor control.

The vagus nerve stimulator is a device, like a pacemaker, with an electrode coiled around the left vagus nerve, and a small, subcutaneous pulse generator placed in the left thoracic area [Swanson et al., 2019; Fallahi et al., 2023]. VNS is minimally invasive, performed as an outpatient [Das, 2011; AANS] or in-hospital procedure. The VNS transmits regular pulses of electrical energy to the autonomic nervous system, including the splanchnic nerve distally (efferents) and the brainstem proximally (afferents). Patients generally are unaware of the electrical pulses. VNS is well-tolerated with known side effects, often mild and transient, related to focal irritation of the vagus nerve including hoarseness, and coughing [AANS, 2024]. The complication rate is <5% and includes rare postoperative site infections, device failure due to lead fracture, or irritation of the vagus nerve resulting in dysphagia, dysphonia, or even vocal cord paralysis. Rarely, the device is not well-tolerated, and requires removal [Aalbers et al., 2015; Revesz et al., 2016].

Fortuitously, there is already a large, multicenter trial in place using VNS to reduce cytokines, including IL-6, in patients with rheumatoid arthritis, “The RESET-RA Study” (NCT #04539964) [Peterson et al., 2024]. Importantly, VNS leads to reduction of inflammatory cytokines (IL-6, TNF-α, IL-1β); the serum levels of cytokines are reduced, or normalized, but not entirely eliminated; therefore, the bioavailability of cytokines, including IL-6, allows the maintenance of competent immunosurveillance, even if the serum levels are undetectable in standard clinical assays [Dalli et al., 2017]. The ongoing trial for treatment of rheumatoid arthritis is a prospective, randomized, double-blind, sham-controlled, multicenter study. Subjects are assigned randomly in a 1:1 ratio to the treatment or control group. Subjects in the treatment group receive active stimulation for 1 min once per day, and those assigned to the control group receive sham stimulation for 1 min once per day. Those assigned to the sham control group have a crossover option at 12 weeks. The stimulation pulse parameters for reduction of cytokines with VNS in the setting of RA are well-defined in terms of frequency, pulse duration, and output current. Ultimately, the electrical parameters could be individualized once a “threshold” level of cytokine is established.

Similar parameters, as used in the RESET-RA study [Levine et al., 2020; Peterson et al., 2024] could be used for the proposed trial in patients with glioblastoma as the objective is to reduce systemic inflammation, the “inflammatory reflex,” and lower IL-6 to non-detectable levels or barely detectable, normal (<5–10 pg/mL) levels, which can be influenced by age, gender, depression or stress [Sullivan et al., 2018; Bremner et al., 2020]. By contrast, in patients with glioblastoma, blood levels of IL-6 are significantly elevated, between 50 and 100% higher than healthy individuals, with a three-fold higher probability of GBM development with a cut-off value of 19.8 pg/mL [Jarmuzek et al., 2023] [Table 2].

### 3.1 Implications of the hypothesis- changing the current paradigm

**Fig. 4:**
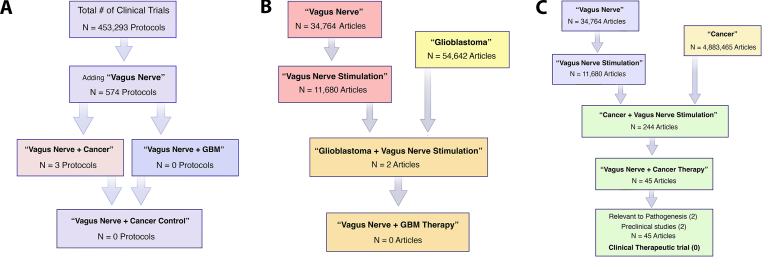
A ClinicalTrials.gov search (Fig. 4A) on 6.25.2023, updated on 7.22.24 reveals no prior or current clinical trials of VNS targeting IL-6, or other cytokines, for cancer control, including glioblastoma. There were three trials (2023) and currently seven trials (2024) studying inflammation, quality of life (fatigue, insomnia), or neuropathic pain using VNS for non-CNS cancer. A PubMed® search (Fig. 4B) revealed no prior articles in 2023 focusing on glioblastoma and VNS focusing on cytokines (e.g., IL-6) as a target; likewise there were only 4 articles linking “vagus nerve” to “cancer”, with none focused on a clinical trial for cancer control (Fig. 4C). See text for details.

To the best of our knowledge, the cytokine (IL-6) reduction by use of VNS represents an entirely novel approach for treatment of glioblastoma, or systemic cancer. The hypothesis was first presented at a satellite meeting of the Society of Neuro-Oncology (SNO-SSA) [Brem, 2023] and remains an innovative concept, confirmed by a search of two databases (July 22, 2024), ClinicalTrials.gov [Fig. 4A] and PubMed Central® [Fig. 4B and C]. The database, ClinicalTrials.gov is the most comprehensive registry of clinical trials worldwide spanning all 50 states and 221 countries [Kowalczyk et al., 2022]. There exist multiple protocols for vagus nerve stimulation (VNS) in patients with cancer designed to mitigate the systemic symptoms of inflammation, e.g., fatigue (NCT0453013), radiation induced (NCT03553485) or postoperative (NCT05743530) inflammation. None, however, are phase II trials focused on cancer control, treatment of glioblastoma, or immune modulation (Fig. 4A), without the standard oncological endpoints of tumor response, progression-free survival or overall survival. There have been no clinical trials with VNS, in combination with checkpoint blockade, despite over 3000 clinical trials designed to evaluate immune modulation of T cells [Robert, 2020]. Likewise, using PRISMA guidelines [Page et al., 2021], a PubMed® (search on 6.15.23), using the term “vagus nerve” yielded 34,764 results; combining “vagus nerve” with “glioblastoma” resulted in only 2 articles, one dealing with intraoperative, electrophysiological monitoring of a patient with a glioblastoma [Schekut'ev et al., 1996], the other focused on seizure control in a patient with a VNS who subsequently developed a glioblastoma [Chrastina et al., 2015]. An updated PubMed® search (7.22.2024) revealed again only two papers using the index words of “glioblastoma” and “vagus nerve stimulation” [Chrastina et al., 2015; Kumaria and Ashkan, 2023] and none using pro-inflammatory cytokines (e.g., IL-6) as a target (Fig. 4B), and none using VNS in combination with cytokine reduction and/or ICI. Likewise, a PubMed search revealed only 4 articles linking “*vagus nerve*” to “*cancer*”, with none focused on a clinical trial for cancer control (Fig. 4C), and an updated search (7.22.24) again reveals no reports of a clinical trial using VNS for cancer control. If validated, the current hypothesis that cytokine (IL-6) reduction through recruitment of VNS, and an allostatic control of the immune system, would represent a major paradigm shift. The proposed clinical trial links classic neuroscience, updated with modern electronic neuromodulation of the cholinergic ‘inflammatory reflex,’ integrating immuno-oncology with the emerging field of cancer neuroscience [Amit et al., 2024; Bernstock et al., 2024; Park and Lee, 2024; ].

It is intriguing that within the current toolbox of neurosurgery, there already exists a potentially powerful tool, VNS, to reduce the inflammaging, mitigate the SASP, and normalize the glioma microenvironment to convert immune resistance, a “cold” tumor, to one that responds to the body's own immune system. Alternatively, cytokine reduction by VNS could synergize with next-gen immunotherapy in the form of a combination therapy with ICIs, cytokine reprogramming (in combination with inhibitors of inflammatory cytokines, or agonists that enhance efficacy of T cells, e.g., IL-2, IL-12, CD40), oncolytic viruses, CAR T cell, or dendritic cell vaccines [Uslu et al., 2024]. Based on preclinical studies [Tsukamoto et al., 2018; Lamano et al., 2019; Yang et al., 2021], reduction of IL-6 is more effective with concomitant ICI. An isolated use of IL-6 blockade could potentially increase the expression of other immunosuppressive cytokines or upregulate PD-1/PD-L1, but given in combination (VNS + ICI), would predictably represent an effective combination.

### 3.2 Combination with current immunotherapeutic approaches

An advantage of minimally invasive, surgically implanted VNS is that it is well-tolerated as an implant in patients with epilepsy, becoming more effective with the passage of time [Uthman et al., 2004]. Therefore, VNS could be used long-term to convert glioblastoma, and other forms of cancer, into a chronic, manageable disease, with cytokine reprogramming of the immune system, thereby enabling T cells to effectively eliminate glioma cells. It is conceivable that activating the immune system with VNS could eradicate glioblastoma with either a brief induction period, analogous to the long-term effects in CAR T cell therapy after a single infusion [O'Rourke et al., 2017; Finck et al., 2020], or the effects of a single dose of an ICI on the immune microenvironment in melanoma that can predict clinical outcomes [Huang et al., 2019]. Alternatively, cytokine reduction to effect long-term, benefit to patients harboring a glioblastoma might require sustained stimulation, which is well-tolerated based on the experience with patients using VNS for medically resistant epilepsy. It would be ideal to evaluate the dose scheduling in an animal model, but that is challenging as most of the preclinical models for VNS are acute experiments [Datta-Chaudhuri et al., 2021]. Current models are constrained because they are open-loop systems, with electrodes tethered externally to the mouse [Datta-Chaudhuri et al., 2021]. With further device miniaturization, it is conceivable to produce a chronic, animal model, more reflective of human application. In addition, there are the inherent problems of modeling immunotherapy in mouse models as the individual tumor may not recapitulate the genomic, mutational landscape of the highly heterogeneous, human glioblastoma, or the complex, immune microenvironment [Haddad et al., 2021; Frederico et al., 2023]. However, in either mouse models or cell culture systems, the immune landscape upon IL-6 blockade changes in a manner expected to increase the efficacy of checkpoint therapy [Tsukamoto et al., 2018; Kubli et al., 2021].

### 3.3 VNS for treatment of Non-CNS cancers (lung, breast, G.I., skin, etc.) and metastatic cancer

It is also conceivable that VNS could be applied to multiple types of cancers (melanoma, lung cancer, etc.) where ICI is currently effective (metastatic melanoma, colorectal cancer, non-small cell lung cancer, renal cell carcinoma) but only transiently, while durable responses are limited in 80% of these patients due to resistance to immune checkpoint inhibitors [Berland et al., 2024].

Another testable implication of the hypothesis is that VNS could be used for prevention or treatment of metastatic cancer as IL-6 is linked to the metastatic process [Knüpfer and Preiss, 2007; Bonapace et al., 2014; Fukumura et al., 2016; Erin, 2020; Raškova et al., 2022; Rozenberg et al., 2023; Song et al., 2024; Ray et al., 2024], using serum IL-6 levels as a “theranostic” biomarker [Jiang et al., 2000; Knüpfer and Preiss, 2007; Laino et al., 2020; Ma et al., 2023]**.** Furthermore, there exists the potential to combine VNS/cytokine (IL-6) reduction with CAR T cell [Bagley et al., 2024a], dendritic cell [Liau et al., 2023; Song et al., 2024], or anti-VEGF therapy (bevacizumab) [Saidi et al., 2009; Fukumura et al., 2016; Incio et al., 2018]**,** with the aim to alter the TME from an immunologically “cold” to an immune responsive environment.

There has been much interest recently in the interaction of the CNS with systemic cancer, mainly at the peripheral tissue and organ level [Phillips et al., 2021; Hanahan and Monje, 2023; Winkler et al., 2023; Mancusi and Monje, 2023; Restaino et al., 2023; Park and Lee, 2024; Bernstock et al., 2024; Amit et al., 2024; Swanton et al., 2024]. Provocative questions include: *i*) does the brain monitor incipient cancer throughout the body [Gidron et al., 2005]; *ii*) is there a specific brain “connectome” where the vagus nerve relays information ?[Hachem et al., 2018; Ottaviani et al., 2022; Jin et al., 2024], i.e., neural networks that regulate systemic, tumor immunity [Reijmen et al., 2018], does the vagus nerve relay information on inflammation influenced by levels of cytokines, such as IL-6, in the serum or in the tumor microenvironment?; *iii*) could tumor-treating fields and other “electroceutical” approaches [Stupp et al., 2017; Kumaria and Ashkan, 2023] work by affecting the autonomic nervous system, the vagus nerve, or cytokines, such as IL-6?; *iv*) is there an “*immunological homunculus*” in the brain [Thayer and Sternberg, 2009] that controls cytokines, immunity and cancer development, similar to the control of motor movement or autonomic functions such as breathing and heart rate?; and finally, *v*) are cancer metastases under the control of the central and peripheral nervous systems [Shi et al., 2013], mediated by IL-6 [Haldar et al., 2023]; if so, could VNS thwart cancer metastases as is proposed here for glioblastoma initiation, progression, and infiltration throughout the brain? The recent finding that peripheral immune events strongly activate the body-brain, vagal-based circuits to inform the brain of emerging inflammatory responses [Jin et al., 2024] offers an exciting new framework to modulate immunotherapy for cancer and is consistent with the proposed use of VNS to control cancer within and outside of the brain, targeting pro-inflammatory cytokines, primarily IL-6.

Ricon-Becker et al. [2021], in a study of patients with breast cancer awaiting surgery, suggested another mechanism by which vagal activity could improve cancer prognosis by activating monocytes as antigen presenting cells (APCs), to enhance tumor antigen presentation, thereby facilitating T-cell immune surveillance, as well as enhancing MCH-1 expression via IFNɣ on monocytes [Ricon-Becker et al., 2021]. IL-6 expression leads to immune resistance, in part, by systemic dysregulation of conventional type-1 dendritic cell, cDC1 [Bear et al., 2020]. Restoring dysfunctional (“exhausted”) T cells or dysfunctional dendritic cells is an area of active research [Datsi and Sorg, 2021; Linette and Carreno, 2022; Singh et al., 2022; Friedrich et al., 2023; Andersson and Tracey, 2012; Espinosa-Carrasco et al., 2024] and could be used in concert with vagal nerve stimulation. A specific example is the use of CD40 agonists as costimulatory molecules to activate dendritic cells [Singh et al., 2022] which Yang et al. [2021] found to be synergistic models in combination with IL-6 targeting, prolonged survival and eradicated gliomas in preclinical models.

In support of the hypothesis, preliminary work suggests that VNS could have a positive effect to harness the immune system and contribute to cancer control at multiple sites in the body beyond the CNS. Reijmen et al. [2021], in a preclinical lung tumor model, found that VNS stimulated lymphocytic infiltrates into tumors, enhanced the activation of intratumoral CD8^+^ T cells by upregulating IFN-γ and CD137, activation of dendritic cells, arresting the proliferation of MDSCs, and attenuating the inflammatory cytokine, TNF-α. In a small cohort of patients, tissue analysis was more informative than immune monitoring of peripheral blood; taken together these findings support further research to use VNS to optimize tumor response rates in patients with cancer [Reijmen et al., 2021]. Of note, these authors were able to stimulate the VNS briefly and transiently, using a transcutaneous (non-invasive) method of VNS, did not evaluate different stimulation parameters (frequency, duration, strength) and did not use ICIs, – a key component of the integrated approach to cancer immunotherapy linked to the current hypothesis. Also, the authors focused on the cytokine TNF-α, but not IL-6.

Electrical stimulation for cancer therapy, including VNS, is emerging as an increasingly relevant approach to cancer therapy, due to the ability of the vagus nerve to activate important elements of cellular immunotherapy [Das et al., 2022]. The neural innervation of multiple cancers (prostate, G.I., lung), including nerve growth factor, and the dynamic interactions between nerve innervation and cancer progression provides an extraordinary opportunity for clinical translation [Hutchings et al., 2020] but also there is the caveat that the inverse correlation between innervation and outcome of these tumors, vagal stimulation could be a double-edged sword, and theoretically even spur growth [Hutchings et al., 2020]. However, Hutchings et al. [2020] acknowledge they did not examine the key role of cytokines, or the effect of VNS on the immune system.

## 4 Future directions: challenges and opportunities

Furthermore, the extraordinary safety record of VNS in humans after 25 years of FDA-approval and more than 125,000 surgeries worldwide [Peterson et al., 2024], without a report of cancer progression in either within or outside of the CNS, make it unlikely that VNS would promote cancer. As with many cancer therapies that could be a “double-edged sword,’ it would be prudent to have a “safety-run” in the proposed clinical trial to exclude the possibility of cancer progression or pseudo-progression. As proof of principle, Zhang et al. [2021] noted that the use of non-invasive, electroacupuncture (EA) stimulation of the vagus nerve (VNS) in mouse models of breast cancer at the ST36 acupoint reduced proinflammatory cytokines (IL-1β and TNF-α), reducing inflammation locally, and enhanced antitumor immunity, measured by the proportion and cytolytic function of CD8^+^ T cells and NK cells, along with a decline in the accumulation and immunosuppressive activities of MDSCs. In addition, c-fos expression in ChAT + neurons of the dorsal motor nucleus of the vagus increased following electroacupuncture; as a control, the benefit of EA was blocked by subdiaphragmatic vagotomy.

VNS could be used long-term, implanted once, cost-effective, and avoid long-term drug, autoimmune, or financial toxicity. If necessary, due to battery failure or other complications, VNS can be revised surgically [Aalbers et al., 2015], but this has been uncommon in the established use of VNS chronically for treatment of epilepsy or depression [Uthman et al., 2004; Revesz et al., 2016; Simpson et al., 2022]. Promising noninvasive treatments, such as transcutaneous stimulation of the vagus nerve, are in development [Bonaz et al., 2016; Baig et al., 2022; Reijmen et al., 2021; Das et al., 2022; Divani et al., 2023] but at the risk of decreased efficacy [Simpson et al., 2022]; nearly all patients undergoing VNS use established, minimally invasive, neurosurgical techniques. An area of future progress could be selective pharmacological modulation of the vagus nerve using cholinergic (e.g., GTS-21), ion channel complexes, or adrenergic agents, – but these approaches, still in development, carry the usual constraints of pharmacokinetics, pharmacodynamics (off-target effects) toxicity, and drug resistance. [Van Maanen et al., 2009; Bonaz et al., 2016; Chatterjee et al., 2017; Hutchings et al., 2020; Zhang et al., 2021; Das et al., 2022; Vishwakarma et al., 2022].

In addition, a recent review, focusing on the potential of non-invasive VNS, cancer progression and immunotherapy discussed the above findings, supporting the hypothesis presented here, but significantly, emphasized *non-invasive VNS*, described enthusiastically as a “hidden treasure” [Abdullahi et al., 2023]. These authors, mechanistically, did not focus on cytokines, such as IL-6, but instead discussed oxidative stress via SIRT1, and the role of the hypothalamic-pituitary-axis as well as release of corticosteroids from the adrenal gland in addition to the cholinergic “inflammatory reflex” [Abdullahi et al., 2023]. The authors note that VNS could provide additional clinical benefits: reduction of lymphopenia, improvement of fatigue, and enhanced quality of life [Abdullahi et al., 2023]. Because of decreased morbidity of non-invasive VNS, it could be especially useful in the elderly population [Abdullahi et al., 2023]. Another hypothetical mechanism for the potential efficacy of VNS to treat glioblastoma, would be for VNS to interfere with glutamine signaling [Kumaria and Ashkan, 2023], the predominant excitatory neurotransmitter of the brain, shown to promote glioma invasion and growth [Venkataramani et al., 2019].

Non-invasive VNS relies on the cutaneous distribution of vagal afferents, either at the external ear (auricular branch of the vagus nerve) or at the neck (cervical branch of the vagus nerve) [Abdullahi et al., 2023; Abdullahi et al., 2024]. The use of auricular branch or other non-invasive approaches to VNS could be a future, testable approach through clinical trials should the primary hypothesis presented here, involving direct stimulation of the vagus nerve with an implantable stimulator, prove to be effective in modulating the immune response and/or halting tumor progression. The distinction is important between external, non-invasive, auricular, and cervical VNS compared to a direct, minimally invasive, VNS with an implant [Vessell et al., 2024, Cicutti et al., 2024]. For example, auricular (non-invasive) stimulation of the vagus nerve is ineffective for treatment of active rheumatoid arthritis [Baker et al., 2023], in contrast to minimally invasive, neurosurgical approach, possibly because the minimally invasive approach is in greater proximity to the site of immunological activity [Baker et al., 2023] as well as activating both the efferent and afferent fibers of the vagus nerve.

The link of the autonomic nervous system, mediated by the vagus nerve, and the cholinergic influence on T cells, places cancer in the scope of a potentially new “dysautonomia” [Goldstein et al., 2002] supporting a view of allostasis [Lee, 2019] rather than the classical notion of homeostasis. The implication of the hypothesis is to view glioblastoma specifically, and neoplasia in general, as a “dysautonomia” [Goldstein et al., 2002; Goldstein, 2021; Goldstein, 2022; Goldstein, 2024], a disorder of the “immune-stat” [Goldstein, 2022] mediated by the vagus nerve activity, through cholinergic, α7nAchR (α7nicotinic acetylcholine receptor) and catecholaminergic, adrenergic receptors, including α2-adrenergic receptors that have strong anti-tumor activity in animal tumor models, including ICI-resistant models [Zhao et al., 2023] with β2-adrenergic receptors on CD4^+^ T cells [Bonaz et al., 2016; Guyot et al., 2019]; *ii)* hematopoietic cells, (lymphocytes, monocytes, myeloid-derived suppressor cells), mobilized to the glioma, fueled by the SASP, with elevated levels of IL-6; and *iii)* reduction of systemic cytokines, especially IL-6, using VNS as an “*electroceutical*”. VNS is proposed to convert the glioma TME to an immunostimulatory environment, supporting activated T cells to thwart tumor growth, preventing progression and recurrence. If the hypothesis is valid, and it can be evaluated in a randomized, controlled, clinical trial, then the concepts can be extended to multiple other cancers that are resistant currently to ICIs or vaccine therapy. Table 3 outlines the questions and proposed measures to harness the potential of reprogramming the immune system for treatment of intracerebral and systemic cancer through re-engineering of an established neurosurgical procedure: vagus nerve stimulation.

## 5 Conclusions

If we can therapeutically reset the cytokine balance, (specifically, IL-6), then we could suppress inflammation, the SASP, and with it, control cancer. The prevention of glioblastoma recurrence [Fig. 2], by itself, is associated with long-term survival [Hertler et al., 2023]. The concept of “cancer without disease” [Folkman and Kalluri, 2004] of maintaining dormancy [Brem et al., 1976; Naumov et al., 2008] was proposed decades ago, by suppressing neovascularization, one of the hallmarks of cancer [Hanahan, 2022]. It is conceivable that we could achieve a similar, modern objective of “*cancer interception”* [Domchek and Vonderheide, 2024] by suppressing tumor-associated inflammation and normalization of IL-6 that is not only central to the pathogenesis of glioblastoma, multiple other forms of cancer, as well as diverse neurological illnesses [Pons-Espinal et al., 2024; Robert et al., 2023]. The testable hypothesis presented here of cytokine reduction mediated by VNS, combined with ICI, demonstrates the synergy of two systems – the CNS and the immune system – working in concert could conceivably control malignant progression within the brain and at other sites in the human body.

## Funding statement

This work did not receive any funding.

## CRediT authorship contribution statement

**Steven Brem:** Writing – review & editing, Writing – original draft, Visualization, Validation, Supervision, Conceptualization.

## Declaration of competing interest

The author declares that he has no known competing financial interests or personal relationships that could have appeared to influence the work reported in this paper.

## Data Availability

No data was used for the research described in the article.
